# Concerning Myxœdema

**Published:** 1887-07

**Authors:** Rudolph Virchow


### CONCERNING MYXŒDEMA.

ABSTRACT OF REMARKS BY PROFESSOR RUDOLPH VIRCHOW BEFORE THE BERLIN MEDICAL SOCIETY.

[Translated by Professor Elbert Wing from the Original Report in *Deutsche Medizinische Wochenschrift*, No. 6, 1887.]

PROFESSOR VIRCHOW had this subject assigned for discussion, not so much because he personally had a great number of observations to report, as for the reason that the question had not received that amount of attention in Germany which its importance merits. The Clinical Society of London appointed a commission to investigate this disease, which reported, toward the close of October, 1886, 110 cases. Since that date Riess, in Germany, has reported a case which he previously observed, and recently, and in consequence of that report, Erb has reported two cases which were under observation in his clinic. As in the majority of the English cases, these three are without autopsies, but they show that such cases occur among us, and that we must begin to take part in the work which such an important subject demands.

Until recently the thyroid gland has been classed among the so-called blood glands, not so much because a great deal was known of its influence upon the blood, as because it was concluded that so large an organ must have an important influence; and since the gland has no duct, it was assumed that its product passed through the blood, and so the thyroid was compared not only with the spleen but also with the thymus gland. However, when a fundamental investigation of the histology of the organ was made, it was quickly seen that there were essential differences between the thyroid and lymphatic glands, as samples of which the thymus gland particularly, and the spleen also in a certain sense, are considered.

There is not the least analogy in any of the lymph glands, to the large alveolar spaces which in the normal thyroid gland are almost filled with cells. Professor Virchow had formerly instituted a parallel between the tissue of the thyroid gland and the cortical layer of the supravenal capsule and the hypophysis cerebri (pituitary body). There are analogies in structure between these three bodies, and they are particularly marked between the hypophysis and the thyroid.

Recently one of the most acute observers of the younger school in England, Horsley, upon the basis of experimental as well as anatomical investigations, has again advanced the old opinion. He claims that the thyroid gland is a hæmatopoietic organ. Professor Virchow has seen his preparations, and must acknowledge that in the stuma of the gland he has seen certain colony-like collections of lymphoid elements which, *cum grano salis*, however, may be compared with the malpighian bodies of the spleen. Horsley is of the opinion that this substance has power to exert some special influence over the mixture of the blood, and he has made countings of the blood from the arteria and vena thyroidia, which have shown an increased number of white blood corpuscles in the vein. Professor Virchow doubts that this is the rule. The quantity of this tissue mentioned is so minimal in the preparations of Horsley that one can find numberless other territories of the body which contain much more lymphoid tissue. When one assumes that colorless blood corpuscles are brought into the circulation from the thyroid gland, it must be only a minimum in contrast with the many other sources from which the blood constructs its elements.

In regard to the clinical aspect of the question, attention was first called to it in 1878 by Ord, one of the physicians at

St. Thomas' Hospital in London. The term "myxœdema" was originated by him. This name is based upon a striking symptom of the disease, a gradual hypertrophy of certain supecficial tissues, especially those of the face. A peculiar elastic fullness of the cheeks occurs, and the lips become more and more prominent, and sometimes also the alæ nasi. Occasionally the process extends to the neck. In severe cases similar appearances are seen on the extremities. Occasionally appearances occur which have a similarity as to anasarca, with an anasarca which proceeds from above downwards. Myxœdema presents transformations to forms which are described as pachydermatous, and it has really happened that Charcot gave the name "cachexie pachydermique" to the case which he described.

Ord had the good fortune to be allowed by his patient to excise a piece of the skin, and later an autopsy was held, by means of which it was thought the nature of the disease was discovered, since, in contrast to the watery fluid of ordinary dropsy, which contains a moderate quantity of albuminates, a fluid was found which contained mucin. Now ordinary fatty tissue has the property in many cases, of undergoing a change into a gelatine-like mass, in which mucin also occurs. This condition is found in the human body in cases of atrophy of the fatty tissue, in which through other circumstances, a reduction in the volume of the tissues is prevented. Professor Virchow, therefore, at first concluded that the same thing occurs in myxœdema, a metaplasia of subcutaneous fat with a transformation into mucous tissue. However, since during a recent sojourn in London, he has had an opportunity to examine the preparations there, he thinks his first conclusions are incorrect. In the discussed cutaneous and subcutaneous sections there is a marked growth of the connective tissue, consequently the theory of a passive process is excluded, the process is of an irritative character, it is allied to the new formations, and resembles in certain respects the inflammatory processes. It is remarkable that this process is almost completely lacking upon the surface of the skin, but the deeper layers of the skin and the upper layers of the subcutaneous tissue are involved. However, it is not the fat cells of the subcutaneous tissue which show the hypertrophy, but the bands of connective tissue which penetrate between the lobes of fat. This process confirms, to a degree, the theory of the old authors expressed in the name Leucophlegmasia, and it approaches in fact the Pacchydermia in Charcot's sense, in which such a hypertrophic process of the sub-cutaneous tissue plays a prominent part.

The preparations shown Professor Virchow in London, were not able to demonstrate where the mucin is present. When one looks carefully he can see numerous processes of mere growths in the tissue which belong to the connective tissue, with the appearance of the growth of mucin. Professor Virchow is therefore not entirely persuaded that the growth of mucin in myxœdema plays so important a part as the name implies. In this connection the researches of Horsley have indeed produced further discoveries. He extirpated the thyroid gland of monkeys, and according to his declaration, and according to the analysis of Halliburton, a chemist exceptionally qualified, in his (Horsley's) opinion, there resulted in consequence of this procedure a condition, called by him, mucinoid, in which the blood contained mucin; then resulted therefrom a sort of mucinous dyscrasia. While the blood usually contains no mucin, he found, in the blood of a monkey which lived 55 days after the operation, 0.35 per thousand, and in the blood of one which lived 49 days, 0.08 per thousand.

Horsley further found that after extirpation of the thyroid gland the quantity of mucin furnished by the salivary glands was greatly increased, and that the parotid gland produced a secretion which contained more and more mucin. The skin, tendons and muscles, and the tissue of the parotid and submaxillary glands themselves, were examined, and in all cases an increase of contained mucin was found.

Horsley therefore draws the conclusion that the thyroid gland is a kind of regulator of elementary changes, and especially controls the transformation of certain elements in the final disintegrative processes, which, when it is removed, ceases, and now the albuminates remain in a state of mucin, and do not proceed to the final disintegrative processes. If these researches, that is, the chemical researches and all that belongs with them, are confirmed, there will thereby probably be afforded an explanation of the nature of the swellings which appear upon the outer parts of the body. One must however conclude that the swelling is not, as Horsley himself seems to think, a simple condition of retention, but also an active irritant process.

But the manifestations of myxœdema ever present only one link in the chain of phenomena of the disease, and which is indeed upon the first glance the most striking. The list of other manifestations is still more impressive, and among these are those of a purely nervous nature. Sir William Gull had, five years before Ord, described five cases occurring in women, who during life were presumed to be in a cretinoid condition. It is hardly doubtful that these were cases of myxœdema. The cases observed since that time have shown, that while the external appearances of the affected individuals change in a characteristic manner, a number of changes appear in the nervous system, especially in the way of mental ability, which, upon the whole, appear to be those of depression, and which occasionally lead to complete idiocy. The affected individuals remain in a peculiar condition, take no interest in their surroundings, have apparently no mental ability, when spoken to sharply, reply, indeed, but in a very much altered voice, protruding the tongue, but the reply always indicates a brain working in a normal direction, but very feebly and very slowly. From this list of changes the question comes prominently forward, just what is the relation between myxœdema and other disturbances of a nervous nature. Horsley has observed quite closely analogous conditions in monkeys in which he had removed the thyroid gland. In these experimental researches he endeavored to observe very carefully the course of the manifestations, and as a result divides the course of the disease into three stages. He differentiates first a nervous stage, in which the nervous manifestations are markedly prominent, among which a peculiar tremor and occasionally cramp-like manifestations occur; then, a second, a mucinoid stage; and finally a third, an atrophic stage. In this connection it is to be observed that the animals were able to live only a relatively short time. At once there appeared a marked diminution of the temperature which rapidly progressed, so that it was only by means of artificial heat that the animals could be kept alive longer than the usual period of five to seven weeks. There was an opportunity given, in these animals, to approach nearer the question as to what is the nature of the process in the brain in these cases. The suggestion that there is some nerve connection by which changes in the cerebral cortex are brought about, naturally occurs at once.

The operations were always performed under the strictest aseptic conditions in order to surely protect the greater nerves, and the anatomical investigation of the nerves failed to show anything which could be considered a softening worthy of mention, consequently Horsley concluded that the brain suffered disturbances since the vitiated blood no longer furnished the proper nourishing material.

The question of the relation of goitre and cretinism has from early times been constantly recurring and discussed. And the fact is, that endemic cretinism does not exist anywhere in the world where endemic goitre is not also present. On the other hand sporadic forms of cretinism and of goitre have also repeatedly been observed. And further, we cannot say that goitre and cretinism stand in any certain parallelism in the same individual. The most marked strumous forms are as a rule associated with integrity of the mental faculties, or at least with no degree of disturbance bance of those faculties which can be compared with cretinism, and again there are very many cretins who have no marked goitre.

About fifty years ago there was a royal commission in Sardinia which carefully traveled through the goitre and cretin district. It found that among the cretins which they examined 2,011 were not strenuous, while 3,912 had goitre. It is of course not to be denied that the examination in the living is often difficult to make, but still it remains from these observations that in or about a third of these cretins had no goitre worth mention. Also in these cases the necessity of the combination did not exist. Nothing in this relation was more clearly marked than congenital struma. Since cretinism is established during fœtal life, one is forced to conclude that when struma and cretinism are present in the same individual, one must find in congenital struma disturbances which indicate cretinism. So far as earlier literature is concerned, and in his own experience (Geschwülste III, p. 54) such a combination has not occurred. Nevertheless it cannot be denied that a very common form of cretinism exists which undoubtedly has a very great similarity with myxœdema. In Italy and especially in Sardinia where there is such a large number of cretins, the common people have for centuries differentiated these diseases. The one they call "cretins," from creta, chalk, and the others "marrons." Professor Virchom has been to the trouble to investigate this terminology, and has concluded, that by the term cretin, the leucophlegmatic cretins are meant; and this form of cretinism has undoubtedly very great similarity to myxœdema.

These appearances resemble another congenital condition, the so-called congenital rachitis, which has no resemblance to the acquired rachitis. Professor Virchow demonstrated two preparations, two new-born children, in which these appearances were present in a marked degree. In both cases the thyroid gland was exposed, and it was shown, that at least in the case of that child which presented the most marked appearances of myxœdema, the thyroid gland was present in that condition in which one would expect it to be in a new-born child. On the contrary, the second child, which died at a much earlier stage, had an extreme atrophy of the thyroid gland. A complete and original absence of the thyroid must be the greatest exception. Professor Virchow possesses only one preparation in which one-half of the thyroid gland is lacking, but this is by no means an absence of the entire gland. Many children with congenital rachitis—disregarding the disturbances of growth of bone in the extremities—present very marked changes at the base of the brain, which correspond with certain changes in cretins, namely, synostosis of the basilar bones and atrophy of the hypophysis.

A real support for the significance of the reported observations has been won through the recent experience with the so-called cachexia strumapriva. Just recently Küster has presented such a case to the Society, and the existence of such a peculiar neurotic cachexia can be confidently asserted. Upon the ground of this fact it is already recognized as a necessity in surgery, not to extirpate the entire gland, but to leave at least a part of it. Horsley found that after a partial extirpation, the rest of the gland very rapidly increased in size, and that thus a compensatory hypertrophy occurred.

In this connection Basedoni's disease, the cachexia ophthalmica, with its retroaction upon the heart, the brain and the eyes, is to be remembered.

Professor Virchow closed his remarks with the repeated suggestion, that all of these remarkable processes possess a pressing warning, to keep a careful watch upon the condition of the thyroid gland, also in the general practice of medicine, in order to bring the greatest possible amount of material to the settlement of the question.

